# The Impairment of Blood-Brain Barrier in Alzheimer’s Disease: Challenges and Opportunities with Stem Cells

**DOI:** 10.3390/ijms231710136

**Published:** 2022-09-04

**Authors:** Adolfo López-Ornelas, Adriana Jiménez, Gilberto Pérez-Sánchez, Citlali Ekaterina Rodríguez-Pérez, Alejandro Corzo-Cruz, Iván Velasco, Enrique Estudillo

**Affiliations:** 1 División de Investigación, Hospital Juárez de México, Mexico City 07760, Mexico; 2 Hospital Nacional Homeopático, Hospitales Federales de Referencia, Mexico City 06800, Mexico; 3 Laboratorio de Psicoinmunología, Instituto Nacional de Psiquiatría Ramón de la Fuente Muñiz, Calzada México-Xochimilco 101, Colonia San Lorenzo Huipulco, Tlalpan, Ciudad de México 14370, Mexico; 4 Laboratorio de Neurofarmacología Molecular y Nanotecnología, Instituto Nacional de Neurología y Neurocirugía Manuel Velasco Suárez, Mexico City 14269, Mexico; 5 Laboratorio Traslacional, Escuela Militar de Graduados de Sanidad, Secretaría de la Defensa Nacional, Batalla de Celaya 202, Lomas de Sotelo, Miguel Hidalgo, Ciudad de México 11200, Mexico; 6 Instituto de Fisiología Celular—Neurociencias, Universidad Nacional Autónoma de Mexico, Mexico City 04510, Mexico; 7 Laboratorio de Reprogramación Celular, Instituto Nacional de Neurología y Neurocirugía Manuel Velasco Suárez, Mexico City 14269, Mexico

**Keywords:** Alzheimer’s disease, blood-brain barrier, MSCs, NSCs

## Abstract

Alzheimer’s disease (AD) is the most common neurodegenerative disorder and its prevalence is increasing. Nowadays, very few drugs effectively reduce AD symptoms and thus, a better understanding of its pathophysiology is vital to design new effective schemes. Presymptomatic neuronal damage caused by the accumulation of Amyloid β peptide and Tau protein abnormalities remains a challenge, despite recent efforts in drug development. Importantly, therapeutic targets, biomarkers, and diagnostic techniques have emerged to detect and treat AD. Of note, the compromised blood-brain barrier (BBB) and peripheral inflammation in AD are becoming more evident, being harmful factors that contribute to the development of the disease. Perspectives from different pre-clinical and clinical studies link peripheral inflammation with the onset and progression of AD. This review aims to analyze the main factors and the contribution of impaired BBB in AD development. Additionally, we describe the potential therapeutic strategies using stem cells for AD treatment.

## 1. Introduction

Alzheimer’s disease (AD) is the most common cause of dementia; it is a progressive cognitive and functional impairment with memory loss that affects over 40 million people worldwide [1]. AD incidence is expected to increase in the coming years; most cases are sporadic and of late-onset, without presenting a proven hereditability [2]. The prevalence increases with life expectancy and affects 10–30% of people over 65 years of age [3]. AD displays a complex etiology that represents a great challenge to elucidate the mechanisms that trigger its onset. However, multiple research groups have provided important insights regarding the biological mechanisms that cause AD.

The most prevalent hypothesis about the onset of AD is the deposition of Amyloid-beta (Aβ) fragments that induce neurotoxicity and, ultimately, neuronal death [4]. However, another possible cause widely accepted is the accumulation of neurofibrillary tangles (NFT) derived from the hyperphosphorylation of Tau [5], a protein that organizes cytoskeletal elements of neurons. Although Aβ and Tau alterations have a significant role in AD, its complexity implicates other essential factors that contribute to its manifestation [6].

Recently, it has been proposed that alterations related to AD include the disruption of the blood-brain barrier (BBB) [7,8] and the activation of the innate immune system, which has an essential role at its onset and progression [9]. Here we mainly analyzed the role of BBB impairment and how this can be used to treat AD.

## 2. Results

### 2.1. The Role of Aβ in AD

Amyloid precursor protein (APP) is a transmembrane protein with three domains, extracellular, transmembrane, and intracellular [10]. Soluble APP (sAPP) is involved in cell survival, neurite growth, synaptogenesis, and synaptic plasticity [11,12]. APP can be cleaved by several secretases and the non-amyloid processing involves α-and γ-secretases. In amyloidogenic forms, β-secretase (BACE1), rather than α-secretase, is the main protease involved, producing mostly Aβ1-40 and, to a lesser extent, the more amyloidogenic form Aβ1-42. Aβ1-40 tends to accumulate in the vasculature, while Aβ1-42 constitutes the predominant form in amyloid plaques [13]. Monomers, oligomers, fibrils, and amyloid plaques are the different conformations of Aβ aggregation in the brains of patients with AD. Aβ does not adopt a single folded form but acquires a set of conformations prone to aggregation in the form of Aβ oligomers, and these are transient forms between monomers and fibrils [14]. Oligomers and fibrils are the most toxic arrangements for forming Aβ [15]. There is no clear correlation between Aβ deposits and the onset of AD [16], but the temporary emergency appears to be well defined. Neuroimaging studies indicate that Aβ accumulation begins in cerebral areas with high metabolic activity [17]. In the first stage, deposits can be found in the temporal, frontal, and occipital lobes. In the second stage, they cover the areas of isocortical association, except in the primary sensory-motor areas, and some are located into the parietal lobes and the hippocampus begins to be affected. In the final stage, they encompass primary isocortical areas, the striatum, thalamus, and hypothalamus [18].

Importantly, ε4 allele of apolipoprotein E (*APOE*ε4) is the main genetic risk of late-onset AD. Apo-E proteins participate in Aβ clearance and Apo-E4 is less efficient in performing this process than other isoforms such as Apo-E3, suggesting that its participation in Aβ accumulation is in accordance with the increased deposition of Aβ observed in the brain of *APOE*ε4 allele carriers [19].

AD pathology investigation has been widely focused on Aβ theory, however, there are still no successful therapies to treat AD by targeting Aβ and recently the theory was challenged since some inconsistencies were found in the research of Aβ*56 isoform [20]; therefore, the role of Aβ*56 in AD should be carefully considered and other strategies such as targeting Tau or microglia activity need to be addressed as therapeutic alternatives to Aβ targeting [21].

### 2.2. Tau and AD

Tau neurofibrillary tangles are closely related to neuronal loss and clinical symptoms of AD [16]. Although Aβ can initiate a cascade of events related to AD onset, Tau impairment is probably the main effector that induces neurodegeneration. Tau is involved in microtubule stabilization and dynamics, myelination, axonal transport, neurogenesis, neuronal excitability, glucose metabolism, DNA protection, iron homeostasis, motor, learning, and memory functions [22]. The accumulation of Aβ is a hallmark of AD, while Tau pathology also exists in other tauopathies [23]. Tau is predominantly expressed in the adult human central and peripheral nervous system, with different isoforms in the central nervous system (CNS) produced through alternative splicing of the Microtubule-associated protein tau (MAPT) comprising 16 exons located on chromosome 17q21.3 [24]. Tau is most abundant in the axons of nerve cells. In the brain, Tau has several post-translational modifications and the most studied is its phosphorylation which negatively regulates its ability to interact with microtubules. Tau is primarily an intracellular protein, but it is also present in the extracellular space and interstitial fluid [25]. Tau is phosphorylated in the AD human brain forming intraneuronal aggregates known as NFT [26], although not all phosphorylated Tau is aggregated [27]. In AD, Tau aggregates follow a well-defined pattern, which begins in the entorhinal cortex and hippocampus [25]. Tau pathology can also be mediated by Aβ oligomerization which induces Tau oligomerization in vitro [28]. Moreover, Aβ deposition was associated with increased phosphorylated Tau in the cerebrospinal fluid from AD patients and the 5xFAD mouse model [29].

### 2.3. The Synergy between Aβ and Tau

Aβ depositions and NFT are hallmarks of AD and act through different mechanisms to induce neurotoxicity. Nevertheless, therapies that target Aβ plaques or Tau do not have satisfactory results [30]. Interestingly, Tau is a mediator of Aβ plaques cytotoxicity [31] and Aβ and Tau may interact via intermediate molecules (e.g., some kinases) [32]. An interplay between Aβ and Tau amplifies toxic effects rather than a mode of interaction [33]. The interplay between Aβ and Tau has been suggested in AD pathology. Studies carried out with Tau in its isolated form of human samples with or without Aβ plaques showed that Tau aggregation properties were enhanced in Tau derived from subjects containing Aβ plaques. Moreover, the crossbreeding of APP/PS1 and rTg4510 mice increased Tau deposition about three times [34]. In the cortex and hippocampus from APP/PS1 and cortex tissues from AD patients, Aβ plaques correlated with Tau hyperphosphorylation at the sites S199/S202, which is mediated by CDK5 and CK2 kinases [35]. GSK3β is also involved in Tau hyperphosphorylation in neurons exposed to Aβ. Reciprocally, it has been described that Aβ toxicity depends on Tau phosphorylation, since the inhibition of CDK5 and GSK3β protects neurons from toxic effects exerted by Aβ [36].

Aβ induces tau hyperphosphorylation which generates neurotoxicity. In AD, several kinases, including A-kinase, C-kinase, cyclin-dependent kinase-5 (CDK-5), CaM kinase II, glycogen synthase kinase-3β (GSK-3β), and MAPKs [32,37] lead to hyperphosphorylation of Tau, resulting in its dissociation from MTs and the formation of NFT [38]. Hyperphosphorylated Tau increases cytoskeletal proteins and affects axoplasmic transport, causing neuronal degeneration [39].

Also, Tau facilitates Aβ toxicity in a Tau-dependent manner. For instance, Fyn (a member of the Src-family) phosphorylation is promoted by Aβ, and together with phosphorylated Tau are transferred to postsynaptic membrane receptors, facilitating the interaction with postsynaptic proteins and leads to excitotoxic downstream signaling [40,41].

Aβ plaques and Tau synergistic interaction also affects intracellular targets (e.g., mitochondria), thus amplifying neurotoxic effects. Mitochondrial alterations are implicated in AD [42] and Aβ plaques, NFT, and neurotoxicity are related with mitochondrial alterations [43]. Several proteins interact with Aβ plaques and phosphorylated Tau (e.g., Dynamin-1-like protein Drp1 (DNM1L), caspase-3, HSD17B10), leading to mitochondrial fragmentation and neuronal death [30,44]. Finally, Aβ and Tau coexist in pathological sites in AD [30,45].

### 2.4. Neuroinflammation in Alzheimer’s Disease

In recent years, the sustained immune response in the brain of patients with AD has been considered a substantial part of the central pathology of AD [46,47,48,49,50], which is observed in post-mortem brains and AD preclinical models [51,52,53,54,55,56]. Sustained activation of microglia in the brain and other immune cells has been observed, which exacerbates Aβ and Tau pathologies and could be a link in the pathogenesis of AD [57].

Neuroinflammation is an inflammatory response within the brain and spinal cord [58]. Infections, toxins, and other injuries activate acute inflammation in the brain. Chronic neuroinflammation occurs when there is a lack of balance in the anti-inflammatory and proinflammatory response, as with AD, which presents activated microglia and cytokines [59,60]. Neuroinflammation is not unique to AD and is also seen in Parkinson’s disease (PD) [61,62], chronic traumatic encephalopathy [63], amyotrophic lateral sclerosis [64], and multiple sclerosis [65].

At first, it was thought that the chronic inflammation found in the brain of patients with AD was a response to neuronal loss. However, a chronic immune response (from middle age onwards) [66] is associated with neurodegeneration and Aβ and Tau pathologies, suggesting neuroinflammation as a link between AD and peptide alterations [67,68,69]. Activated microglia surrounds Aβ plaques and NFT in post-mortem samples of AD patients, suggesting its participation in Aβ clearance since microglia can bind soluble and insoluble forms of Aβ through SCARA1, CD, and Toll-like receptors. However, activated microglia by Aβ in AD can also promote the release of proinflammatory cytokines such as TNFα, also produced by astrocytes and neurons [17].

Equivalently, it is proposed that activation of the innate immune system is essential at the onset and progression of AD. Several genes that code for receptors with an immunological role, such as *CD33*, *CLU*, *CR1*, and *TREM2*, are altered in AD [70,71,72]. Various genes that regulate the immune system inside and outside the CNS, such as *BLNK*, *GRN*, *HEXB*, *PYDC1*, *SYK*, and *SLC2A5,* are related to this disease [73,74]. These changes in gene expression suggest a role of the central and peripheral immune systems in AD.

### 2.5. The BBB Barrier in AD

Three brain barriers protect and maintain the brain microenvironment: the arachnoid barrier, the blood-cerebrospinal fluid barrier, and the BBB [75,76].

The BBB is an interface between the peripheral circulation and the CNS. It is a semi-permeable structural and chemical barrier, highly selective, that separates the circulating blood from the brain, and the extracellular fluid [77]. The neurovascular unit is the fundamental component of the BBB and is composed of capillary endothelial cells (its main structure), astrocytes, pericytes, neurons, and tight junctions [78]. In a healthy brain, the BBB does not allow the entrance of several immune cells, hydrophilic molecules, and large proteins into the brain and vice versa [79]. The permeability of BBB is not associated with the molecular size itself: glucose and amino acids pass through the BBB more easily than many ions [80], and permeability increases in the presence of cerebral edema, brain tumors, ionizing radiation injury, inflammation, and other pathological conditions, allowing certain toxic substances to enter brain tissue and ultimately resulting in damage to the CNS [81]. Strokes, immunoneurological diseases, and multiple system atrophy significantly alter the BBB function [82]. However, not all substances are harmful; some antibodies reach injured regions of the brain and contribute to its restoration [83]. It is noteworthy to highlight that many plasma proteins can reach the healthy brain mainly through Clathrin transport [84], thus suggesting the presence of plasma-derived biomolecules with regenerative properties that could be tested to treat ailments such as AD.

A large body of evidence indicates that the BBB is impaired in neurodegenerative conditions [85] such as AD and PD. AD patients have shown a compromised BBB since leakage of plasma proteins and blood cells have been found in brain regions such as the prefrontal cortex, entorhinal cortex, and hippocampus of post-mortem tissues when compared with control brains [85,86,87,88]. Imaging studies further support the disruption of the BBB on AD even at early stages [89]; in line with these data, brain endothelial cells derived from induced pluripotent stem cells (iPSCs) of AD patients display aberrant properties such as high permeability and altered expression of tight junctions [90]. Additionally, animal studies have provided consistent results since rodent models of AD also display a disrupted BBB [91]. Remarkably, rodent AD models suggest an increased vulnerability of BBB to be compromised after an inflammatory insult; therefore, inflammation could contribute to BBB disruption [91].

Excessive Aβ generation and its deposition in the brain also contribute to the disruption of BBB [92] by increasing the damage to the neurovascular unit [8]. Increased BBB permeability in AD could be related to pericyte loss possibly mediated by MMP-9 in ApoE4 carriers. Aβ deposition also is related with pericyte death and the disruption of astrocytes end feet and arterioles coupling. Furthermore, Aβ plaques and NFTs are associated with thickening of cerebral blood vessels leading to hemorrhages [93]. Vascular basement membrane thickening in AD is observed in brain regions such as the cerebral cortex, hippocampus, and thalamus mainly in the parenchymal basement membrane suggesting the participation of astrocytes since they are associated with the synthesis of this basement membrane. Furthermore, other studies reported the altered synthesis and degradation of basement membrane components (e.g., collagen IV) [94,95]. Increased monocyte trafficking through the BBB in response to Aβ has been identified in the peripheral circulation or brain parenchyma and associated with the pathophysiology of Aβ-related vascular disorder [96]. In addition, Aβ induces the expression of vascular adhesion molecules, promoting leukocyte adhesion and transmigration during the pathological process of AD [97]. In animal models it has been shown that Aβ deposits were able to increase the chemoattraction and permeability of neutrophils and bone marrow-derived microglia as a cellular mechanism for the restriction and elimination AB deposits [98,99]. Additionally, it was observed that monocytes were attracted only to the luminal wall of veins with Aβ deposits where they engulf Aβ for its elimination. This process occurs mainly in the cortex and hippocampus [100].

**Figure 1:**
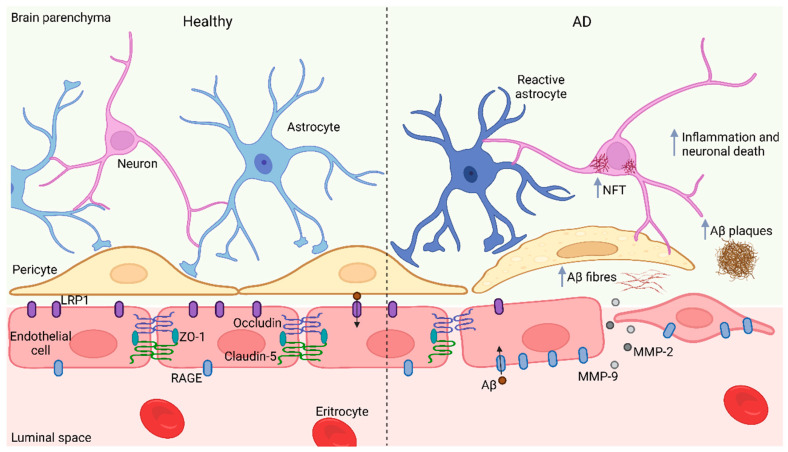
BBB impairment in AD. In normal conditions, levels of RAGE and LRP1 in brain blood vessels are low and high, respectively; however, in AD, blood vessels display opposite levels of these Aβ clearance proteins leading to Aβ accumulation and deposition. Moreover, Aβ decreases the expression of Occluding, Claudin-5 and ZO-1 in brain endothelial cells and increases MMP-2 and MMP-9 activity, thus enhancing BBB permeability. Additionally, Aβ accumulation induces Tau aggregation, inflammation, and neuronal death. BBB, blood brain barrier; AD, Alzheimer’s disease; RAGE, Receptor for advanced glycation end product; LRP1, Low-density lipoprotein receptor related protein 1; Aβ, Amyloid β peptide; ZO-1, Zonula occludens; MMP-2, MMP-9, Matrix metalloproteinase 2 and 9; NFT, Neurofibrillary tangles. Figure created with Biorender.

BBB damage induced by Aβ results not only from direct toxicology in endothelial cells, astrocytes, and pericytes but also from indirect neuroinflammation and oxidative stress triggered by Aβ accumulation, as well as from the impaired interaction between different cellular components in BBB [93]. Moreover, Aβ1-42 decreases the expression of tight junction proteins occluding and claudin-5 in endothelial cells and related tight junction proteins such as ZO-1; conversely, Aβ1-42 increases MMP-2 and MMP-9 activity that subsequently enhances endothelial permeability. Interestingly, the inactivation of the receptor for advanced glycation end products (RAGE) attenuates these impairments, indicating that RAGE mediates the endothelial cell alterations derived from the exposure to Aβ1-42 (Figure 1) [101].

RAGE is the major Aβ influx receptor that promotes Aβ deposition; its expression is increased in brain endothelial cells of AD patients [8,102] and the cortex of 5XFAD mice [88,101]. On the other hand, low-density lipoprotein receptor-related protein 1 (LRP1) is the primary mechanism for Aβ clearance from the brain, and its expression in brain endothelial cells is reduced in AD patients and animal models [103].

BBB impairment facilitates the entry of undesired systemic factors with the potential to contribute to AD progression by mainly triggering proinflammatory conditions that eventually promote neurodegeneration [104]. In fact, the abnormal influx of Aβ oligomers from the milieu to the CNS due to abnormal high and low endothelial levels of RAGE and LPR1 respectively, could contribute to AD progression [79,80,81]. Nevertheless, it is possible that other biomolecules could display an altered pattern of influx and efflux between the CNS and the periphery. Recent findings indicate that aging impairs the BBB transport of plasma proteins to the brain [84], thus, the entrance of plasma proteins could also be altered in AD. Interestingly, BBB endothelial cells display the highest number of AD-related genes with altered expression, which strongly suggests that their altered function could play a key role in AD pathogenesis [105]. Hence, harmful systemic factors could reach the brain and contribute to AD manifestation due to a compromised barrier function of brain endothelial cells.

Harmful circulating factors that could exacerbate neurodegeneration increase with age. Proteomic analyses revealed that aged blood carries the chemokine CCL11, which is capable of impairing neurogenesis and cognitive performance when administered to young mice; remarkably, antibody blockade of CCL11 rescued the impaired neurogenesis of mice and it would be worth testing whether this strategy is also able to rescue cognitive impairments as well [106]. Further studies identified that aged blood promoted an inflammatory phenotype of brain endothelial cells which was associated with the expression of VCAM1, an adhesion protein upregulated in inflammatory processes; moreover, aged blood contributed to the activation of microglia when administered to young mice [107]. These striking findings demonstrate the deep impact of circulating factors on the propensity to suffer a neurodegenerative disorder, since they promote mainly a proinflammatory profile as individuals age and are capable of altering the BBB.

### 2.6. BBB Impairment: An Opportunity to Treat AD?

In contrast with the harmful agents of the milieu that could reach the brain in AD, the free entrance of protective factors to the brain parenchyma due to a compromised BBB in AD could also be possible; hence, their capability to cross the BBB should be further evaluated and considered as a therapeutic strategy. Some of these factors could be present in blood from young individuals since pre-clinical studies have reported the recovery and improvement of AD mice after receiving plasma from younger animals [108]. Unfortunately, there are still no beneficial effects detected in AD patients after receiving plasma from young volunteers [109]. The divergence of these results could be explained by different causes such as the fact that mice AD models do not entirely reproduce the AD, therefore the animal results could differ from humans. On the other hand, it should also be considered that the effect of beneficial factors could be masked by other unspecific molecules present in plasma. Therefore, there is a need to identify and isolate the specific plasma molecules from human origin with the capability to prevent neurodegeneration in AD.

The identification of beneficial factors in the blood represents a formidable challenge given the extreme complexity of blood which has hampered the identification of especially neuroprotective and rejuvenating cues. Recent pioneering studies identified the protein Clusterin (CLU), upregulated in plasma from exercised rodents and humans. Remarkably, intravenous administration of CLU induced an anti-inflammatory phenotype in the brain endothelial cells of the hippocampus of transgenic AD mice, suggesting its potential as a therapeutic agent to treat this neurodegenerative condition [110]. In line with these findings, another recent work identified the Glycosylphosphatidylinositol specific phospholipase D1 (Gpld1) as a beneficial factor in the blood of exercised subjects that improves cognitive skills and plasticity mechanisms in the brain when transferred to sedentary rodents [111]. Interestingly, there is a correlation between exercise routines and the amelioration of symptoms in AD [112,113]; this information suggests that Gpld1 and CLU should be considered to treat AD since they could be administered intravenously and freely reach the affected areas of the brain. Given this evidence, it is noteworthy to highlight the importance of identifying other blood-borne factors of either young or exercised individuals with neuroprotective properties that could contribute to the improvement of AD treatment.

Although promising, several contrasting reports suggest that BBB remains unaltered in AD and that drugs and blood-borne factors cannot cross it [114,115,116]. If this is the case, there is a need to develop therapeutic strategies to overcome this obstacle. Several biologic molecules, such as antibodies capable of crossing the BBB, have been conjugated with trophic factors and have the potential to treat AD. Detailed and recent information regarding this exciting field can be found elsewhere [117]. However, it is also possible that some neuroprotective factors do not cross the BBB; instead, they could interact and stimulate membrane receptors on brain endothelial cells from the luminal space and initiate beneficial effects by secreting cues to the brain parenchyma. CLU binds to the brain blood vessels when administered intravenously [110] therefore, the possibility that this protein does not cross the BBB cannot be excluded.

A promising strategy to treat AD that has been barely explored and could overcome the obstacle of the BBB is using stem cells as a vehicle of neuroprotective agents. This model will be discussed in the next section.

### 2.7. Cell Delivery of Neuroprotective Factors

**Figure 2:**
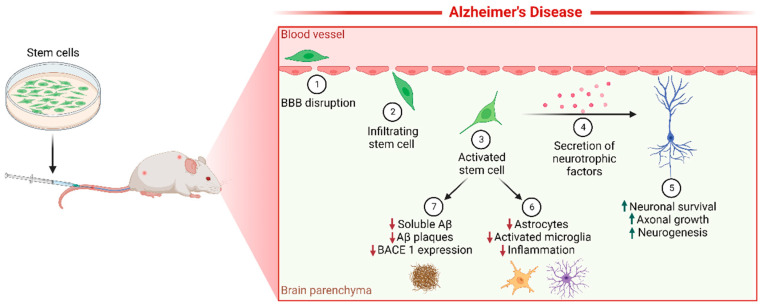
Stem cell-mediated delivery of neuroprotective factors as a model for AD treatment. MSCs and NSCs can cross BBB in response to stimuli produced by neurodegeneration and inflammation such as HGF in AD (1 and 2). Engineered stem cells can secrete neuroprotective factors such as GDNF (3 and 4) and improve neuronal survival, axonal growth, and neurogenesis (5). Furthermore, MSCs decrease proinflammatory mediators, increase anti-inflammatory molecules and reduce Aβ accumulation (6) in animal AD models. AD, Alzheimer’s disease; MSCs, Mesenchymal stem cells; NSCs, Neural stem cells; BBB, Blood brain barrier; HGF, Hepatocyte growth factor; GDNF, Glial derived neurotrophic factor; Aβ, Amyloid β peptide. Figure created with Biorender.

Stem cells can transmigrate through the endothelium and direct toward specific signals such as proinflammatory factors. This homing property has been exploited to evaluate its potential to reach injured areas through low invasive administrations [118,119]. The properties of the microenvironment modified by neurodegeneration and inflammation, along with the BBB permeability can be exploited to treat the disease. One of the strategies proposed is using stem cells (Figure 2) [93,120]

### 2.8. Mesenchymal Stem Cells (MSCs)

MSCs, also known as mesenchymal stromal cells, are multipotent cells and normally can differentiate into adipocytes, chondrocytes, and osteocytes, but under special conditions can also produce neurons; they express growth and neurotrophic factors with effects on axonal growth, neurogenesis, neuroprotection, and neovascularization [121]. They are isolated from adipose tissue, bone marrow, heart, placenta, skeletal muscle, and umbilical cord [122]. MSCs appear to avoid immune rejection given the low expression of the main molecules of the class I histocompatibility complex [123]. MSCs can also reach injured zones of the brain after the induction of an ischemic brain insult when administered intravenously [124]. This migration is possible due to the expression of specific factors that attract MSCs, such as Stromal cell-derived factor-1α (SDF-1α) or Hepatocyte Growth Factor (HGF) [125,126]. Importantly, AD patients display high levels of HGF in serum and cerebrospinal fluid [127,128,129]. Moreover, histologic analysis shows high levels of HGF in the brain parenchyma [104]. These data suggest that the high HGF concentration in the central nervous system could aid MSCs to migrate towards the brain of patients with AD and cross their impaired BBB [130,131]. However, the MSCs biodistribution represents a drawback since they are mainly distributed in other undesired organs such as lung, spleen, heart and kidney. Additionally, the trackability of MSCs could also be a challenge since all the imaging techniques have limitations for their in vivo tracking such as tissue thickness, tracer leakage or short life of radiolabeled tracers [132]. Thus, further efforts must be performed to overcome these issues.

Despite their therapeutic potential per se, MSCs can also be modified to express neuroprotective factors to counteract the neurodegenerative processes produced by AD and halt its progression [133]. Previous works aimed to enhance the therapeutic effects of MSCs through genetic modifications. MSCs have been engineered to express multiple trophic factors such as Placental Growth Factor (PIGF), Glial Derived Neurotrophic Factor (GDNF), Brain-Derived Neurotrophic Factor (BDNF), or HGF in order to increase their therapeutic effect on AD [134,135].

MSCs intravenously injected have benefits in animal models of AD in both prophylactic and treatment paradigms [136,137]. The effects reported include the decrease of soluble Aβ-42, Aβ plaques, γ-secretase activity, and BACE1 expression. Also, inflammation is reduced, since astrocytes and activated microglia decrease, along with iNOS levels, Cox2 expression, and proinflammatory cytokines IL-1 and TNFα. On the other hand, cell death in the hippocampus declines with an increase in anti-inflammatory cytokine IL-10, TGFβ, and enzymes that degrade Aβ [137,138,139,140].

However, with the intravenous delivery of MSCs, some administration hazards remain since few cells reach the target sites; on the other hand, although intra-arterial administration of MSCs could represent an alternative to intravenous administration, this strategy increases the risk of embolic events [141,142].

### 2.9. Neural Stem Cells

Neural stem cells (NSCs) are multipotent cells that give rise to the nervous system; therefore, they are present during development and in adult human individuals in the hippocampus and the subventricular zone (SVZ) [143,144,145], although some authors suggest the absence of NSCs in the adult brain [146]. These cells are obtained directly from brain tissue, from neuronal differentiation of somatic cells that were genetically reprogrammed to pluripotency (iPSCs) [147,148], or from pluripotent embryonic stem cells (ESCs) [149,150]. Their use in regenerative medicine has been widely studied and their application in neurodegenerative diseases has shown encouraging results [151].

For example, NSCs transplantation alleviates animal models of AD [152], replacing damaged neurons and differentiating into neurons or glial cells, and releasing trophic factors related to differentiation [153,154,155]. NSCs can rescue synaptic density [154] and the secretion of neurotrophic factors and cellular restoration improve memory [156]. In addition, some NSCs that overexpress some Aβ-degrading enzymes reduce their aggregation and improve synaptic density [157].

Remarkably, NSCs also exhibit CNS homing capabilities [158,159,160]. They can reach sites lesioned by ischemic insults when administered intravenously and improve motor skills in lesioned rats [161,162], suggesting the potential of these cells to also move toward the brain of patients with AD when administered intravenously. Moreover, NSCs can also reach the injured spinal cord in response to SDF-1α and HGF [163,164]. Engrafted NSCs can migrate, proliferate, and differentiate into cholinergic neurons in AD models [165,166], increasing synapses and improving cognitive function; however, Aβ plaques persist. Similarly, human NSCs overexpressing Choline acetyltransferase (ChAT) and grafted in AD animal models improve cognitive and memory functions and retain their homing towards affected brain regions [167]. A subpopulation of highly migrating NSCs and MSCs, was isolated in a recent study [168]. These NSCs migrate better and the expression of podoplanin (PDPN), a molecule related with organ development, cell motility, tumorigenesis, and metastasis [169], is essential for this enhanced homing. This subpopulation had better effects in models of AD and, likewise, in glioblastoma. Remarkably, the overexpression of PDPN showed an increase in migration.

Interestingly, NSCs can be modified to express therapeutic agents to treat certain diseases. Previous research groups have engineered NSCs with an inducible expression system to treat gliomas [159,170,171]. Moreover, engineered NSCs have also been used to treat neurodegenerative diseases [172].

A problem with AD is the poor migration of NSCs to the amyloid plaques, which hinders their use as a vehicle for therapeutic molecules for this disease [173]. However, intranasally administered NSCs survive, migrate and differentiate primarily to neurons in the transgenic APP/PS1 mouse model, with relatively limited glial differentiation, a high proportion of differentiation to cholinergic neurons, and decrease Aβ accumulation by upregulation of degrading enzymes, along with reductions in neuroinflammation, cholinergic dysfunction, and pericyte and synaptic loss. Notably, an increase in neurogenesis was also observed in the adult hippocampus with favorable cognitive changes [174].

On the other hand, there are several drawbacks that need to be addressed in order to use NSCs in AD; for instance, there are ethical concerns of fetal-derived cells that are avoided employing adult-derived cells. Notwithstanding, other important limitations of using NSCs are the low availability of donors and the high probability of immune rejection [175].

Significantly, NSCs from ESCs provide limitless donor cells, but they have the inconveniences of immunoreaction because they are allogeneic, therefore, immunosuppressive therapy is necessary. Moreover, it is possible that undifferentiated cells in cultures could give rise to a teratocarcinoma after their transplantation or systemic injection [176].

Ethical controversies surround the use of human ESCs, thus other sources of NSCs are required. The reprogramming of somatic cells led to the obtention of iPSCs by the induction of genes related with pluripotency during embryogenesis [177,178,179]. iPSCs share characteristics with ESCs and can differentiate to NSCs. Using patient-derived iPSCs avoids ethical issues and, theoretically, an immune rejection.

Finally, it is important to highlight that some studies suggest that NSCs share niches and characteristics with cancer stem cells and may be the origin of these malignant cells [180]. However, the study of how NCSs can be tumorigenic continues under investigation [181].

## 3. Conclusions

AD is a progressive neurodegenerative disease with no effective treatment. BBB impairment could be exploited as a gate towards the CNS in order to deliver therapeutic agents more efficiently. Blood-borne factors and stem cells can be an effective option because of their neuroprotective and regenerative potential, but their mechanism of action is still under investigation, although many preclinical studies suggest positive results. A combination of stem cell therapy, whether transplanted or as a vehicle for therapeutic molecules, and the administration of well-identified blood-borne factors could be a complementary therapeutic strategy to the current drugs.
